# The role of gender in the association between mental health and avoidable hospitalization

**DOI:** 10.1093/eurpub/ckac131.496

**Published:** 2022-10-25

**Authors:** A Catalini, C Mazza, C Cosma, L Stacchini, M Caminiti, G Minutolo, D Genovese, G Ferraguzzi, E Cavaretta, F Cedrone

**Affiliations:** 1 Department of Biomedical Sciences and Public Health, University of the Marche Region, Ancona, Italy; 2 School of Public Health, University of Pavia, Pavia, Italy; 3 Department of Health Science, University of Florence, Florence, Italy; 4 Department of Public Health Sciences, University of Perugia, Perugia, Italy; 5 Department PROMISE, University of Palermo, Palermo, Italy; 6 Department of Biomedical Sciences for Health, University of Milan, Milan, Italy; 7 Health Management of “SS. Spirito” Hospital, Local Health Authority of Pescara, Pescara, Italy

## Abstract

**Background:**

Hospital discharge records (HDR) can indirectly assess the quality of primary care through algorithms proposed by the Agency for Health Research and Quality (AHRQ). Mental illnesses or substance addictions represent barriers to accessibility to medical care which, can lead to hospitalizations deemed potentially preventable. The aim of this study is to evaluate the gender differences in the association between potentially preventable acute hospitalizations whether mental health or addiction.

**Methods:**

The study examined HDRs of the Local Health Authority of Pescara, Abruzzo, period 2015-2021. The aggregate Prevention Quality Acute Composite 91 (PQI-91) has been coded according to the indications of the AHRQ. Were selected HDRs with a diagnosis of depression, psychosis, alcohol or substance abuse according to Enhanced ICD-9-CM Elixhauser algorithm. Four univariate logistic regression models were implemented correcting for age.

**Results:**

In the study period 252,775 HDRs of which 3,459 PQI-91 were analyzed. A diagnosis of depression is positively associated with a PQI-91 hospitalization only in the male gender (aOR 3.16; 95%CI 2.18-4.58) and not in the female one (aOR 1.13; 95%CI 0.75-1.72). The same is true for a diagnosis of psychosis, males (aOR 2.41; 95%CI 1.66-3.48) and females (aOR 1.19; 95%CI 0.67-2.12). In both genders there was an association with substance abuse, males (aOR 3.92; 95%CI 2.65-5.81) and females (aOR 2.68; 95%CI 1.19-6.07), while for the alcohol abuse the female gender is positively associated (aOR 2.52; 95%CI 1.11-5.73) and not the male one (aOR 1.24; 95%CI 0.80-1.92).

**Conclusions:**

Gender is an innovative approach to health inequalities: women and men respond to a different diagnostic-prescriptive appropriateness, which depends both on biology and on social, cultural, psychological and economic distances. Research efforts must be made to observe the effect healthcare access disparities have on patients who experience mental illness or addiction.

**Key messages:**

• Mental health can limit access to primary care for other comorbidities or acute diseases; poor-quality primary care can result in preventable hospitalizations that increase the cost of health care.

• The gender differences for the same diagnosis of mental illness/addiction that cause potentially preventable acute hospitalizations impose gender-specific strategies aimed at modifying care pathways.

